# A bundle to prevent postinduction hypotension in high-risk noncardiac surgery patients: the ZERO-HYPOTENSION single-arm interventional proof-of-concept study

**DOI:** 10.1016/j.bjao.2025.100392

**Published:** 2025-04-11

**Authors:** Kristen K. Thomsen, Alina Kröker, Linda Krause, Karim Kouz, Christian Zöllner, Daniel I. Sessler, Bernd Saugel, Moritz Flick

**Affiliations:** 1 Department of Anesthesiology, Center of Anesthesiology and Intensive Care Medicine, University Medical Center Hamburg-Eppendorf, Hamburg, Germany; 2 Outcomes Research Consortium®, Houston, TX, USA; 3 Institute of Medical Biometry and Epidemiology, University Medical Center Hamburg-Eppendorf, Hamburg, Germany; 4 Department of Anesthesiology and Center for Outcomes Research, University of Texas Health Science Center, Houston, TX, USA

**Keywords:** anaesthesia induction, arterial catheter, blood pressure, general anaesthesia, haemodynamic monitoring, hypotension alarm, norepinephrine, propofol

## Abstract

**Background:**

Postinduction hypotension is common and associated with organ injury but might be largely preventable by careful anaesthetic management. We thus aimed to quantify the severity and duration of postinduction hypotension in high-risk noncardiac surgery patients treated with a hypotension prevention bundle.

**Methods:**

In this prospective single-arm interventional proof-of-concept study, 107 high-risk noncardiac surgery patients were treated with a hypotension prevention bundle. The bundle included continuous intra-arterial blood pressure monitoring, a hypotension alarm set at a mean arterial pressure (MAP) of 75 mm Hg, careful administration of anaesthetic drugs, and continuous administration of norepinephrine when MAP decreased below 75 mm Hg. The primary endpoint, AUC65, was derived from a plot of MAP over time for the first 15 min after induction of general anaesthesia as the area of the plot under a MAP of 65 mm Hg .

**Results:**

Of 107 patients, 55 (51%) had at least one MAP reading <65 mm Hg, but only 16/107 patients (15%) had a MAP <65 mm Hg for at least one continuous minute. Patients had a MAP <65 mm Hg for a median (25% percentile, 75% percentile; minimum–maximum) of 0.2 min (0.0, 0.8; 0.0–5.2 min). The median AUC65 was 0.1 mm Hg . min (0.0, 4.1; 0.0–40.6 mm Hg min).

**Conclusions:**

We observed minimal postinduction hypotension in high-risk noncardiac surgery patients treated with a hypotension prevention bundle. However, randomised trials are needed to confirm that using the hypotension prevention bundle helps reduce postinduction hypotension.

In patients having noncardiac surgery with general anaesthesia, hypotension is common and associated with organ injury.^1^, ^2^, ^3^ In about one-third of instances, hypotension occurs after induction of general anaesthesia but before surgical incision.^4^ Unmodifiable risk factors for this ‘postinduction hypotension’ include older age, male sex, and a high American Society of Anesthesiologists (ASA) physical status class.^5^, ^6^ However, postinduction hypotension is mainly driven by modifiable factors, most importantly by anaesthetic drugs that cause vasodilation.^6^ Anaesthetic-induced vasodilation can be effectively treated with vasopressors such as norepinephrine. It is thus reasonable to assume that careful anaesthetic management will prevent most instances of postinduction hypotension.

We have previously shown that continuous blood pressure monitoring helps clinicians reduce postinduction hypotension.^7^, ^8^ It therefore seems plausible that postinduction hypotension can be further reduced by using a *hypotension prevention bundle* that—in addition to continuous blood pressure monitoring—includes setting hypotension alarms at a threshold to enable early intervention, careful administration of anaesthetic drugs, and continuous administration of vasopressors.

We therefore aimed to quantify the severity and duration of postinduction hypotension in high-risk noncardiac surgery patients treated with a hypotension prevention bundle—combining continuous intra-arterial blood pressure monitoring, a hypotension alarm set at a mean arterial pressure (MAP) of 75 mm Hg, careful administration of anaesthetic drugs, and continuous administration of norepinephrine to prevent hypotension, treat hypotension, or both. We primarily quantified postinduction hypotension as AUC65, which was derived from a plot of MAP over time for the first 15 min after induction of general anaesthesia as the area of the plot under a MAP of 65 mm Hg.

## Methods

### Study design

This prospective single-arm interventional proof-of-concept study was conducted in patients scheduled for noncardiac surgery with general anaesthesia at the University Medical Center Hamburg-Eppendorf, Hamburg, Germany between 7 March and 7 August 2023. The study was approved by the ethics committee on 27 February 2023 (Ethikkommission der Ärztekammer Hamburg, Hamburg, Germany, registration number 2022-100896BO-ff), and all patients provided written informed consent. The study was registered at ClinicalTrials.gov (NCT05842759) on 5 March 2023 (principal investigators: Moritz Flick, Kristen K. Thomsen). We report our study according to the *Transparent Reporting of Evaluations with Nonrandomized Designs (TREND)* statement.^9^

### Patients

We enrolled patients scheduled for elective noncardiac surgery with general anaesthesia who were at least 45 yr old, who were classified ASA physical status class 3 or higher, and in whom intra-arterial blood pressure monitoring with an arterial catheter was planned for clinical indications. We did not include patients who were scheduled for emergency or transplant surgery; who previously had organ transplant surgery; who were septic or pregnant; who had contraindications for propofol administration; and in whom rapid sequence induction or awake fibreoptic tracheal intubation was planned.

### Hypotension prevention bundle

All participating patients were treated with the hypotension prevention bundle. Before starting induction of general anaesthesia, an arterial catheter was inserted in the radial artery for continuous intra-arterial blood pressure monitoring using local anaesthesia and ultrasound guidance. The arterial catheter was connected to a saline-filled pressure transducer system that was zeroed to the level of the right atrium.^10^ A hypotension alarm was set at a MAP of 75 mm Hg on the patient monitor (Infinity Delta; Dräger Medical, Lübeck, Germany). A syringe infusion pump for continuous norepinephrine infusion was prepared and connected to a peripheral or central venous catheter. For induction of general anaesthesia, an opioid and propofol were given, and a neuromuscular blocking agent. Propofol was given only after the clinical effects of the opioid were apparent. Propofol was administered over 90 s at a dose of 1.5 mg kg^−1^ actual body weight in patients younger than 55 yr and at a dose of 1.0 mg kg^−1^ in patients who were 55 yr old or older. The continuous norepinephrine infusion was started to treat hypotension when the MAP decreased below 75 mm Hg, and the norepinephrine infusion rate was then titrated to maintain MAP above 65 mm Hg.

### Blood pressure data

We continuously recorded beat-to-beat blood pressure values. We excluded artifactual blood pressure values using the following rules: (1) values designated as artifacts by study personnel; (2) systolic blood pressure >280 mm Hg or <30 mm Hg; (3) systolic blood pressure lower than diastolic blood pressure plus 5 mm Hg; or (4) diastolic blood pressure <10 mm Hg or >150 mm Hg. For statistical analysis, MAP values were averaged in non-overlapping 10-s episodes. The mean MAP in a 10-s episode was calculated from all available single MAP values within the episode. If all MAP values within a 10-s episode were artifacts, the missing mean MAP was replaced by the mean of the two mean MAP values from the neighbouring 10-s episodes. If all MAP values in two or more consecutive 10-s windows were artifacts, the missing mean MAP values were replaced using the mean MAP values from the nearest neighbouring non-artifact 10-s episode.

### Endpoints

All study endpoints quantifying the severity and duration of postinduction hypotension were assessed within 15 min after induction of general anaesthesia, specifically, within 15 min after opioid administration. Our primary endpoint was the AUC65. We additionally investigated how many patients had at least one MAP <65, 60, 50, and 40 mm Hg and how many patients had a MAP <65, 60, 50, and 40 mm Hg for at least one continuous minute. We also determined for how long patients had MAPs less than 65, 60, 50, and 40 mm Hg. We further assessed the AUC60, AUC50, and AUC40 (all derived similarly tto the AUC65). To evaluate high blood pressure, we derived areas of the MAP vs time plot above thresholds of 100, 110, 120, and 140 mm Hg and refer to these as AAC100, AAC110, etc. The cumulative dose of norepinephrine indexed to actual body weight was also recorded.

### Statistical analysis

We describe patient characteristics, anaesthesia-related data, and haemodynamic data as absolute number (percentage) for categorical data or as median (25% percentile, 75% percentile; minimum–maximum) or mean (standard deviation) for continuous data.

To calculate the area under (or above) the MAP vs time plot for a specified MAP threshold, we first determined the difference between each recorded MAP value and the threshold value (e.g. 65 mm Hg) during 10-s intervals. For values below the threshold, this difference was positive (indicating hypotension), and for values above the threshold, the difference was negative (indicating hypertension). We then multiplied each difference by 10 s to capture the time-weighted impact of each interval. Only positive values (for hypotensive episodes) or negative values (for hypertensive episodes) were summed across all intervals to calculate the total area under or above the threshold. Finally, the resulting sum was divided by 60 to convert the measurement into units of mm Hg x min, providing a measure that reflects both the severity and duration of deviations from the predefined MAP threshold.

We did not perform a formal sample size calculation for this study. Based on previous studies investigating strategies to reduce postinduction hypotension,^7^^,^^8^ we estimated that 100 patients would be sufficient to determine the efficacy of our hypotension prevention bundle. Expecting a drop-out rate of 20%, we planned to enrol 120 patients.

## Results

**Fig 1:**
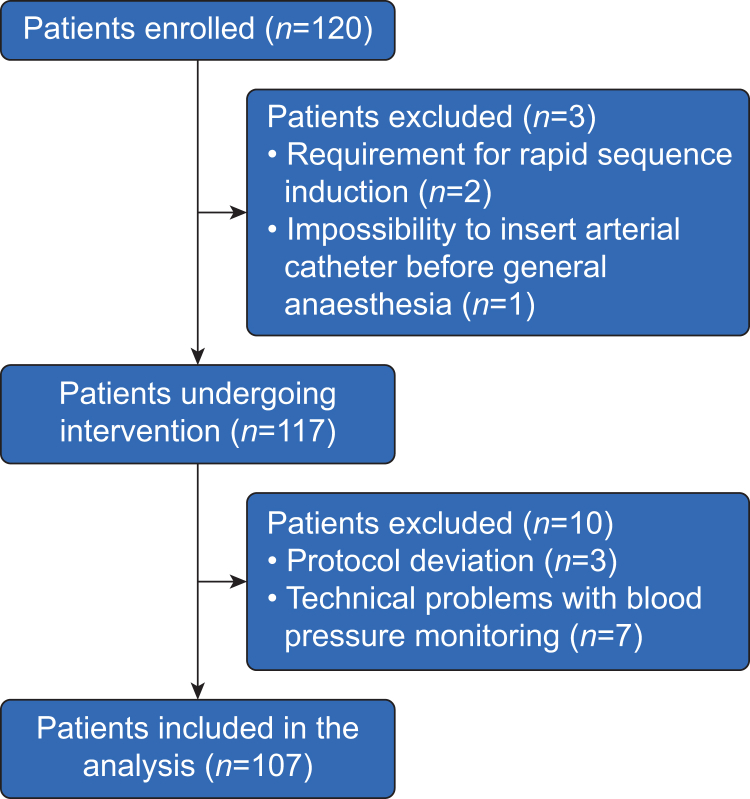
Flow chart illustrating patient screening, enrolment, and reasons for exclusion.

We enrolled 120 patients—but excluded seven patients because of technical problems with blood pressure monitoring. The hypotension prevention bundle could not be applied to an additional six patients, who were therefore excluded: in 2/120 patients (2%), norepinephrine was not administered continuously, and in 1/120 patients (1%), propofol was administered over a shorter time span because of human error. Rapid sequence induction was required in 2/120 patients (2%), and in 1/120 patients (1%) it was not possible to insert an arterial catheter before induction of general anaesthesia (Fig 1). We thus included 107 patients in the final analysis (Table 1).

**Table 1:** Patient baseline and clinical characteristics. Data are presented as median (25% percentile, 75% percentile) or absolute number (percentage). Percentages may not sum up to 100% because of rounding.

Characteristic		Hypotension prevention bundle (*n*=107)
Age (yr)		73 (67, 80)
Height (cm)		173 (167, 180)
Weight (kg)		79 (65, 87)
Sex, n	Female	40 (37)
	Male	67 (63)
American Society of Anesthesiologists physical status classification, *n*	3	102 (95)
	4	5 (5)
*Baseline risk factors, n*		
	Chronic arterial hypertension	99 (93)
	Chronic obstructive pulmonary disease	12 (11)
	Insulin dependent diabetes mellitus	5 (5)
	Chronic heart failure	8 (8)
	Liver disease	6 (6)
	Chronic kidney disease	5 (5)
	Coronary artery disease	27 (25)
	Peripheral artery occlusive disease	38 (36)
	Cerebrovascular disease	30 (28)
Revised cardiac risk index, n	0	20 (19)
	1	45 (42)
	2	31 (29)
	3	10 (9)
	4	1 (1)
*Type of surgery, n*		
	Vascular surgery	49 (46)
	General surgery	13 (12)
	Gynaecological surgery	1 (1)
	Urological surgery	5 (5)
	Trauma/spine surgery	38 (36)
	Others	1 (1)
*Clinical characteristics*		
	Epidural block, *n*	12 (11)
	Total i.v. anaesthesia, *n*	46 (43)
	Balanced anaesthesia, *n*	61 (57)
	Cumulative norepinephrine dose, μg kg^−1^	1.4 (1.0, 1.8)
	Cumulative amount of crystalloid (ml)	250 (100, 400)

Within 15 min after induction of general anaesthesia, 55 patients (51%) had at least one MAP reading <65 mm Hg (Table 2). Sixteen patients (15%) had a MAP <65 mm Hg for at least one continuous minute. Patients had MAPs <65 mm Hg for a median (25% percentile, 75% percentile; minimum–maximum) of 0.2 min (0.0, 0.8; 0.0–5.2 min). The median AUC65 was 0.1 mm Hg x min (0.0, 4.1; 0.0–40.6 mm Hg x min) (primary endpoint). More profound postinduction hypotension was uncommon: 13 patients (12%) had at least one MAP <50 mm Hg. One patient (1%) had a MAP <50 mm Hg for at least one continuous minute (this single episode lasted 1.3 min). Four patients (4%) had at least one MAP <40 mm Hg, but none for at least one continuous minute.

**Table 2:** Summary of areas and durations under thresholds. Continuous data are presented as mean (standard deviation), median (25% percentile, 75% percentile), and range. MAP, mean arterial pressure; sd, standard deviation.

Characteristic	
Area under a MAP <65 mm Hg (mm Hg x min)	
Mean (sd)	3.3 (6.6)
Median (25% percentile, 75% percentile)	0.1 (0.0, 4.1)
Range	0.0–40.6
Area under a MAP <60 mm Hg (mm Hg x min)	
Mean (sd)	1.4 (3.7)
Median (25% percentile, 75% percentile)	0.0 (0.0, 0.8)
Range	0.0–20.2
Area under a MAP <50 mm Hg (mm Hg x min)	
Mean (sd)	0.2 (0.8)
Median (25% percentile, 75% percentile)	0.0 (0.0, 0.0)
Range	0.0–1.6
Area under a MAP <40 mm Hg (mm Hg x min)	
Mean (sd)	0.0 (0.2)
Median (25% percentile, 75% percentile)	(0.0, 0.0)
Range	0.0–1.6
Duration of MAP <65 mm Hg (min)	
Mean (sd)	0.5 (0.8)
Median (25% percentile, 75% percentile)	0.2 (0.0, 0.8)
Range	0.0–5.2
Duration of MAP <60 mm Hg (min)	
Mean (sd)	0.2 (0.5)
Median (25% percentile, 75% percentile)	0.0 (0.0, 0.3)
Range	0.0–2.7
Duration of MAP <50 mm Hg (min)	
Mean (sd)	0.1 (0.2)
Median (25% percentile, 75% percentile)	0.0 (0.0, 0.0)
Range	0.0–1.3
Duration of MAP <40 mm Hg (min)	
Mean (sd)	0.0 (0.0)
Median (25% percentile, 75% percentile)	0.0 (0.0, 0.0)
Range	0.0–0.2

The median AAC100 was 35.3 mm Hg x min (13.5, 109.4; 0.0–492.1 mm Hg x min), and the median AAC120 was 0.1 mm Hg x min (0.0, 10.9; 0.0–221.7 mm Hg x min) (Table 3). Patients were given a median cumulative norepinephrine dose of 1.4 μg kg^−1^ (1.0, 1.8; 0.6–6.1 μg kg^−1^).

**Table 3:** Summary of areas above thresholds. Continuous data are presented as mean (standard deviation), median (25% percentile, 75% percentile), and range. MAP, mean arterial pressure; sd, standard deviation.

Characteristic	
Area above a MAP >100 mm Hg (mm Hg x min)	
Mean (sd)	74.6 (94.2)
Median (25% percentile, 75% percentile)	35.3 (13.5, 109.4)
Range	0.0–492.1
Area above a MAP >110 mm Hg (mm Hg x min)	
Mean (sd)	34.9 (63.9)
Median (25% percentile, 75% percentile)	7.4 (0.1, 37.1)
Range	0.0–342.1
Area above a MAP >120 mm Hg (mm Hg x min)	
Mean (sd)	15.4 (38.7)
Median (25% percentile, 75% percentile)	0.1 (0.0, 10.9)
Range	0.0–221.7
Area above a MAP >140 mm Hg (mm Hg x min)	
Mean (sd)	1.9 (8.7)
Median (25% percentile, 75% percentile)	(0.0, 0.0)
Range	0.0–74.2

## Discussion

We treated high-risk noncardiac surgery patients with a hypotension prevention bundle and observed minimal postinduction hypotension. Although half of the patients had at least one MAP <65 mm Hg within 15 min after induction of general anaesthesia, most episodes with a MAP <65 mm Hg were of short duration. The median AUC65 —that quantifies both the severity and duration of hypotension—was low, specifically 0.1 mm Hg x min(0.0, 4.1 mm Hg x min). An AUC65 of 0.1 mm Hg x min would, for example, result when MAP was 64 mm Hg for 6 s. More profound postinduction hypotension was uncommon: only about 10% of patients had at least one MAP <50 mm Hg, and only one patient had a MAP <50 mm Hg for one continuous minute. Fewer than 5% of patients had at least one MAP <40 mm Hg—and all of these episodes lasted less than a minute.

The burden of postinduction hypotension we observed in this study was substantially less than that we observed in a previous trial of 224 noncardiac surgery patients, showing that continuous intra-arterial blood pressure monitoring helps clinicians reduce hypotension.^8^ In the previous trial, the median (25% percentile, 75% percentile) AUC65 mm within 15 min after induction of general anaesthesia was 46 mm Hg x min (7, 111 mm Hg x min) in patients assigned to intermittent blood pressure monitoring with an upper-arm cuff and 15 mm Hg x min (2, 36 mm Hg x min) in patients assigned to continuous blood pressure monitoring with an arterial catheter (Supplementary Fig. S1).^8^ In contrast, in the present study, the AUC65 within 15 min after induction of general anaesthesia was more than two orders of magnitude smaller than in patients who only had continuous intra-arterial blood pressure monitoring alone in the previous trial (0.1 *vs* 15 mm Hg x min)—although patients in the present study were older and sicker.^8^ It is thus reasonable to conclude that applying a hypotension prevention bundle combining continuous intra-arterial blood pressure monitoring, a hypotension alarm set at a MAP of 75 mm Hg, careful administration of anaesthetic drugs, and continuous administration of norepinephrine is more effective in preventing postinduction hypotension than continuous intra-arterial blood pressure monitoring alone.

We evaluated a bundle of interventions designed to minimise postinduction hypotension. We inserted arterial catheters before starting anaesthetic induction because continuous blood pressure monitoring helps clinicians substantially reduce postinduction hypotension compared with intermittent monitoring.^7^^,^^8^ We set the hypotension alarm at a MAP of 75 mm Hg—a value well above the 65 mm Hg population harm threshold for acute kidney and acute myocardial injury.^11^ Previous studies suggest that hypotension alarms minimally reduce hypotension^12^^,^^13^ and do not improve patient-centred outcomes^14^—perhaps in part because clinicians often do not respond to alarms.^14^ We did not record hypotension alarms and therefore cannot analyse how often clinicians responded to alarms with therapeutic interventions. We gave propofol carefully—over 90 s at a dose of 1.5 mg kg^−1^ actual body weight in patients younger than 55 yr and at a dose of 1.0 mg kg^−1^ in patients who were 55 yr or older. In cohort analyses of older patients, the dose of propofol given during anaesthetic induction was significantly associated with an increased risk for postinduction hypotension.^15^^,^^16^ However, reducing the dose of propofol based on age may risk underdosing. Therefore, we also considered the patient's clinical response during induction and, if needed, patients received additional propofol. Using processed electroencephalography monitoring would have provided additional information on the depth of anaesthesia and optimal propofol doses. Finally, we started a continuous norepinephrine infusion when the MAP was lower than 75 mm Hg and titrated the infusion rate to maintain MAP above 65 mm Hg.^17^

The main limitation of our study is that it was a single-arm interventional proof-of-concept study without a control group. Our results thus reflect the outcomes of a cohort treated with the hypotension prevention bundle but cannot determine the effect of the hypotension prevention bundle compared with routine care. Nevertheless, our results suggest that the bundle we evaluated may help prevent or limit hypotension. Therefore, a formal randomised trial seems warranted to test whether the bundle reduces postinduction hypotension compared with routine care. Trials with large sample sizes would be required to assess the impact of the hypotension prevention bundle on clinical outcomes. Another limitation of our approach is that we cannot determine to what extent each component of our hypotension prevention bundle contributed to hypotension reduction; presumably, each component contributed to some extent. If sufficiently large, the subsequent trial could use a factorial design that would identify the relative contributions of each bundle component.

Using norepinephrine to treat postinduction hypotension risks overtreatment and hypertension.^18^ Vasopressors indeed are independently associated with postoperative acute kidney injury.^19^, ^20^, ^21^ However, we only started norepinephrine when the MAP decreased below 75 mm Hg and most patients were given relatively low doses of norepinephrine, which are unlikely to have detrimental effects on tissue perfusion.^22^ Additionally, hypertension was not a problem in our study and the median values for the AAC100, AAC110, AAC120, and AAC140 were small. In general, while the association between intraoperative hypotension and organ injury is well established,^1^, ^2^, ^3^ the relationship between intraoperative hypertension and organ injury is less clear.^23^^,^^24^

Our department previously conducted several studies on hypotension during anaesthetic induction and surgery that presumably raised clinicians' awareness of hypotension.^5^, ^6^, ^7^, ^8^^,^^25^, ^26^, ^27^ Our results may thus generalise poorly to other institutions. We included high-risk noncardiac surgery patients who were at least 45 yr old. Presumably it is easier to prevent hypotension in healthier patients who are thus less likely to benefit from our hypotension prevention bundle. Our hypotension prevention bundle included interventions designed to treat and prevent the most common causes of postinduction hypotension but did not consider all possible causes of postinduction hypotension—such as bradycardia or myocardial depression caused by anaesthetic drugs.^6^^,^^26^

## Conclusions

In our single-arm interventional proof-of-concept study, we observed minimal amounts of postinduction hypotension in high-risk noncardiac surgery patients treated with a hypotension prevention bundle combining continuous intra-arterial blood pressure monitoring, a hypotension alarm set at a MAP of 75 mm Hg, careful administration of anaesthetic drugs, and continuous administration of norepinephrine to treat hypotension. However, randomised trials are needed to confirm that using the hypotension prevention bundle helps reduce postinduction hypotension.

## Authors’ contributions

Study conception and design: KKT, KK, BS, MF

Acquisition of data: AK

Data analysis: LK

Data interpretation: KKT, AK, KK, CZ, DIS, BS, MF

Drafting of manuscript: KKT, AK, BS, MF

Critical revision of article for important intellectual content: all authors

Final approval of the version to be published: all authors

Agreement to be accountable for all aspects of the work thereby ensuring that questions related to the accuracy or integrity of any part of the work are appropriately investigated and resolved: all authors

## Funding

Support was provided solely from institutional sources, departmental sources, or both.

## Declarations of interest

KKT has received honoraria for consulting and giving lectures from Masimo (Neuchâtel, Switzerland). AK has no conflicts of interest to declare. LK has no conflicts of interest to declare. KK is a consultant for and has received honoraria for giving lectures from Edwards Lifesciences (Irvine, CA, USA). KK is a consultant for Vygon (Aachen, Germany). CZ has no conflicts of interest to declare. DIS has received research funding from Edwards Lifesciences; he also is an advisor and has equity interest in Perceptive Medical (Newport Beach, CA, USA). BS is a consultant for and has received institutional restricted research grants and honoraria for giving lectures from Edwards Lifesciences (Irvine, CA, USA). BS is a consultant for Philips North America (Cambridge, MA, USA) and has received honoraria for giving lectures from Philips Medizin Systeme Böblingen (Böblingen, Germany). BS has received institutional restricted research grants and honoraria for giving lectures from Baxter (Deerfield, IL, USA). BS is a consultant for and has received institutional restricted research grants and honoraria for giving lectures from GE Healthcare (Chicago, IL, USA). BS has received institutional restricted research grants and honoraria for giving lectures from CNSystems Medizintechnik (Graz, Austria). BS is a consultant for Maquet Critical Care (Solna, Sweden). BS has received honoraria for giving lectures from Getinge (Gothenburg, Sweden). BS is a consultant for and has received institutional restricted research grants and honoraria for giving lectures from Pulsion Medical Systems (Feldkirchen, Germany). BS is a consultant for and has received institutional restricted research grants and honoraria for giving lectures from Vygon (Aachen, Germany). BS is a consultant for and has received institutional restricted research grants from Retia Medical (Valhalla, NY, USA). BS has received honoraria for giving lectures from Masimo (Neuchâtel, Switzerland). BS is a consultant for Dynocardia (Cambridge, MA, USA). BS has received institutional restricted research grants from Osypka Medical (Berlin, Germany). BS received honoraria for giving lectures from Ratiopharm (Ulm, Germany). BS was a consultant for and has received institutional restricted research grants from Tensys Medical (San Diego, CA, USA). BS is an Editor of the British Journal of Anaesthesia. MF is a consultant for Lifesciences (Irvine, CA, USA) and has received honoraria for consulting and giving lectures from CNSystems Medizintechnik (Graz, Austria).
