# Supplementary figures and images for: A bundle to prevent postinduction hypotension in high-risk noncardiac surgery patients: the ZERO-HYPOTENSION single-arm interventional proof-of-concept study
Source: BJA Open. 2025 Apr 11;14:100392. doi: 10.1016/j.bjao.2025.100392 (PMC12018566; doi:10.1016/j.bjao.2025.100392)

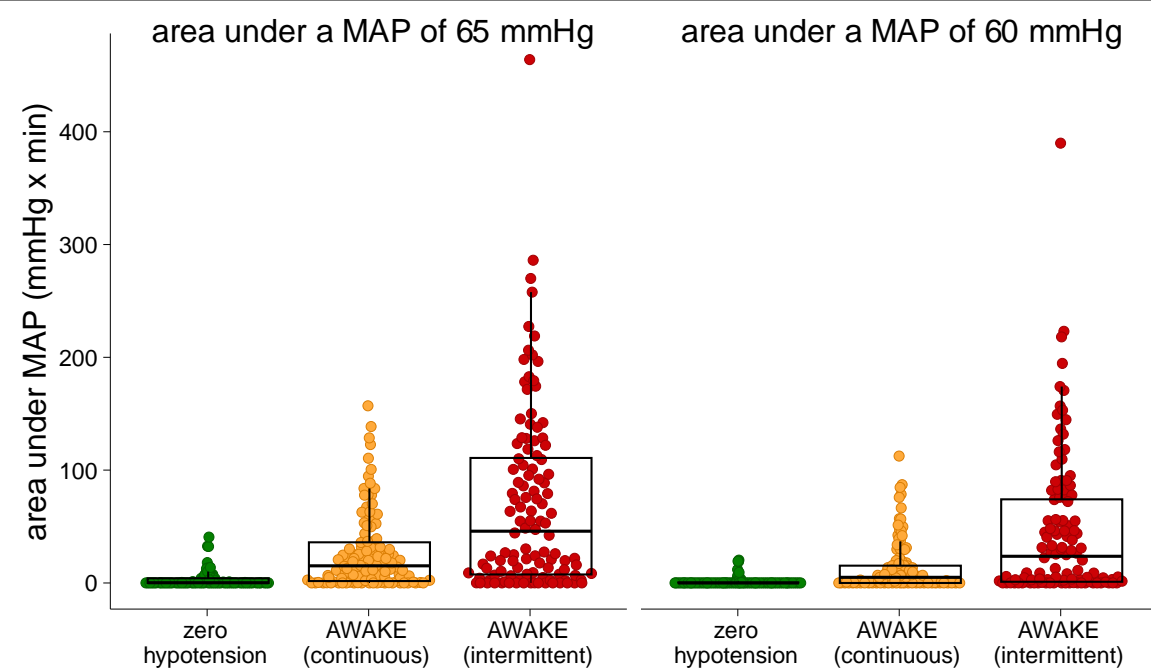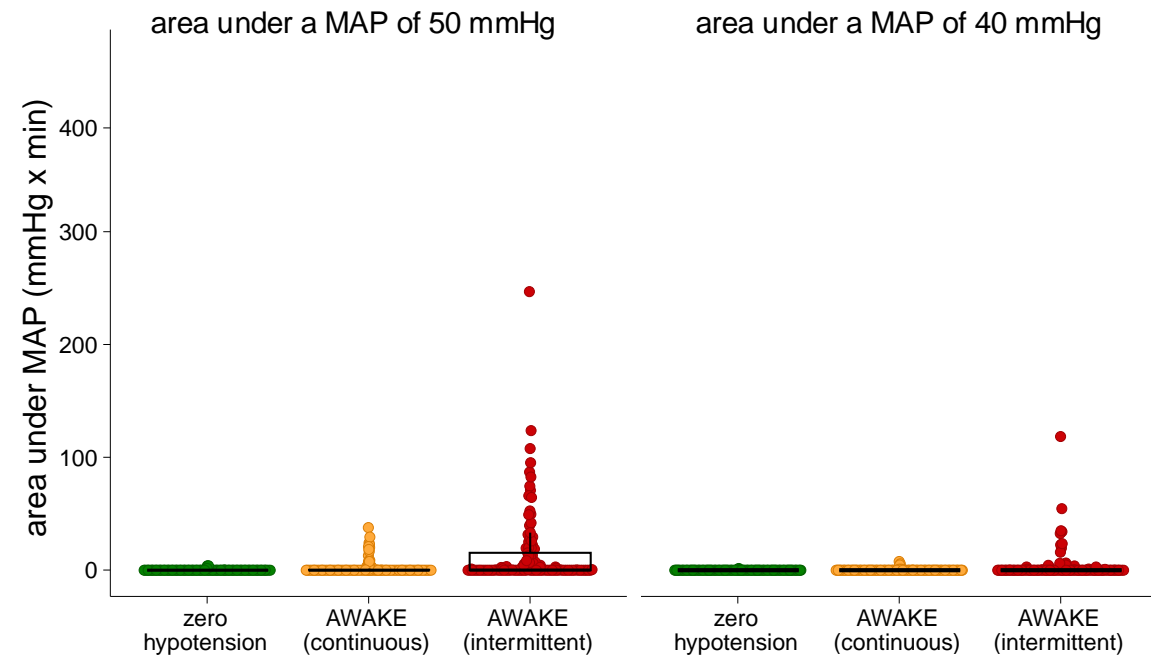

Supplement: Multimedia component 1 [file mmc1.pdf]
